# Electroacupuncture inhibits dendritic spine remodeling through the srGAP3-Rac1 signaling pathway in rats with SNL

**DOI:** 10.1186/s40659-023-00439-0

**Published:** 2023-05-22

**Authors:** Qiaoyun Wu, Chenchen Cai, Xinwang Ying, Yujun Zheng, Jiaying Yu, Xiaoxue Gu, Wenzhan Tu, Xinfa Lou, Guanhu Yang, Ming Li, Songhe Jiang

**Affiliations:** 1 Department of Physical Medicine and Rehabilitation, The Second Affiliated Hospital and Yuying Children’s Hospital of Wenzhou Medical University, 268 Xue Yuan Xi Road, Wenzhou, 325027 Zhejiang People’s Republic of China; 2 Integrative and Optimized Medicine Research Center, China-USA Institute for Acupuncture and Rehabilitation, Wenzhou Medical University, Wenzhou, 325027 Zhejiang China; 3 School of Basic Medical Science, Wenzhou Medical University, Wenzhou, 325027 Zhejiang China; 4 The Wenzhou Key Laboratory for Rehabilitation Research, The Provincial Key Laboratory for Acupuncture and Rehabilitation in Zhejiang Province, Wenzhou, China

**Keywords:** Electroacupuncture, Neuropathic pain, Dendritic spine remodeling, srGAP3/Rac1

## Abstract

Previous studies have shown that peripheral nerve injury can lead to abnormal dendritic spine remodeling in spinal dorsal horn neurons. Inhibition of abnormal dendritic spine remodeling can relieve neuropathic pain. Electroacupuncture (EA) has a beneficial effect on the treatment of neuropathic pain, but the specific mechanism remains unclear. Evidence has shown that slit-robo GTPase activating protein 3 (srGAP3) and Rho GTPase (Rac1) play very important roles in dendritic spine remodeling. Here, we used srGAP3 siRNA and Rac1 activator CN04 to confirm the relationship between SrGAP3 and Rac1 and their roles in improving neuropathic pain with EA. Spinal nerve ligation (SNL) was used as the experimental model, and thermal withdrawal latency (TWL), mechanical withdrawal threshold (MWT), Western blotting, immunohistochemistry and Golgi-Cox staining were used to examine changes in behavioral performance, protein expression and dendritic spines. More dendritic spines and higher expression levels of srGAP3 were found in the initial phase of neuropathic pain. During the maintenance phase, dendritic spines were more mature, which was consistent with lower expression levels of srGAP3 and higher expression levels of Rac1-GTP. EA during the maintenance phase reduced the density and maturity of dendritic spines of rats with SNL, increased the levels of srGAP3 and reduced the levels of Rac1-GTP, while srGAP3 siRNA and CN04 reversed the therapeutic effects of EA. These results suggest that dendritic spines have different manifestations in different stages of neuropathic pain and that EA may inhibit the abnormal dendritic spine remodeling by regulating the srGAP3/Rac1 signaling pathway to alleviate neuropathic pain.

## Introduction

Neuropathic pain is caused by injury or disease of the somatosensory nervous system and can seriously affect patient quality of life [11]. The etiology of neuropathic pain is complex, and it is often difficult to cure clinically [25, 26]. Therefore, it is urgent to understand the pathogenesis and provide a reliable basis for the clinical treatment of this disease.

Dendritic spines are spinous projections on dendritic branches that are the main synapse sites between neurons and play a key role in signal transmission [25, 26]. Dendritic spines can be divided into four types according to their shape: filopodial, thin, mushroom and stubby [10]. Filopodial spines play a role in synaptogenesis and are the beginnings of synaptic connections. Thin spines are immature, with a slender neck and a small head. Mushroom spines are considered mature, with a short, narrow neck and a large bulbous head. Stubby spines have a large head and a fat, short neck and are also mature dendritic spines [18]. Therefore, we can judge local synaptic activity by observing the morphology of dendritic spines, and we can also judge the changes in local synaptic activity by observing changes in the morphological structures of dendritic spines. Abnormal dendritic spine remodeling has been observed in some diseases, including peripheral nerve injury [25, 26], spinal cord injury (SCI) [3] and cutaneous burn [21]. These disease models showed similar patterns of change in dendritic spines, including increased dendritic spine density, increased maturity and closer proximity to the somatic part of neurons. Our previous studies also showed that spinal nerve ligation (SNL) caused plasticity changes of neurons in the dorsal horn of the rat spinal cord, including increased density of dendritic spines [25, 25, 26, 26].

Actin is a cytoskeletal element that is mainly concentrated in the distal end and neck of dendritic spines. As the main structural scaffold, actin gives spines their unique shape [2, 6]. The actin cytoskeleton in dendritic spines plays a role in regulating the maturation of dendritic spines. Moreover, actin is regulated by Rho GTPase (Rac1) [4, 5]. Rac1 is a structural regulator of dendritic spines and maintains a balance between the activated state that is bound to GTP and the inactivated state that is bound to GDP. Rac1 regulates the morphology of dendritic spines by acting on filamentous actin (F-actin) and promotes the aggregation of excitatory AMPA receptors. Studies have shown that stress or injury can promote Rac1 activation [7, 8]. The use of the Rac1-specific inhibitor nsc23766 increased the pain threshold of rats with spinal cord injury (SCI) and chronic constriction injury of the sciatic nerve (CCI) and inhibited abnormal dendritic spine remodeling in the spinal dorsal horn [19, 31]. Furthermore, studies have shown that the N-methyl-D-aspartate receptor 2B subunit (NR2B) was regulated by Rac1 in rats with type-2 diabetic neuropathic pain [4, 5]. Postsynaptic density 95 (PSD-95) is present in the postsynaptic membrane and can interact with NR2B through its specific structure, resulting in NR2B phosphorylation. NR2B knockout reduced dendritic spine density and eliminated Long-term Potentiation (LTP) [13].

Slit-robo GTPase activating protein 3 (srGAP3) is a kind of synaptogenic protein that guides synaptic connections, determines the maturation of dendrites, regulates neuronal progenitors during development, and participates in the early stages of dendritic spine growth [18]. srGAP3 plays a significant role in the initial stage of neuropathic pain [4, 5]. SrGAP3 has three main functional domains, each of which is responsible for the interactions of srGAP3 with other proteins. The GAP domain is Rac1-specific and inhibits Rac1-dependent dendritic spines by accelerating the hydrolysis of GTP [17, 28]. SrGAP3 inhibits Rac1-dependent neurite growth in cerebellar granular neurons. Transfection of srGAP3 without the GAP domain had no such effect on neurite growth [17]. This finding suggests that srGAP3 regulates dendritic spines by regulating RAC1 activity, which is dependent on the GAP domain.

Electroacupuncture (EA) has been used worldwide and has been beneficial for treating many kinds of pain [23, 30]. EA of “Huantiao” and “Weizhong” can inhibit LTP caused by nerve injury and relieve neuropathic pain [16]. Our previous study showed that EA could relieve neuropathic pain by inhibiting dendritic spine remodeling in the spinal dorsal horns of rats with peripheral nerve injury [25, 25, 26, 26], but the specific mechanism remains unclear.

In this study, we examined the morphological structure of dendritic spines in the spinal dorsal horns of rats with spinal nerve ligation (SNL) during different stages of neuropathic pain. Moreover, srGAP3 siRNA and the Rac1 agonist CN04 were used to examine the relationship between srGAP3 and Rac1 and the role of the srGAP3/Rac1 signaling pathway in EA-induced analgesia. The structure of rats is very similar to that of humans in many aspects, including gene composition and biological behavior characteristics. SNL rats show relatively stable neuropathic pain symptoms without losing motor function, and are often used to simulate clinical neuropathic pain diseases [25, 26]. EA has a good analgesic effect in clinical practice. In order to explore its mechanism of action, we made a model of neuropathic pain in rats, and selected therapeutic acupoints and EA frequency based on clinical experience, traditional Chinese medicine theory, anatomical structure of human body and rats, and previous studies, which are as close as possible to the occurrence, development and treatment of clinical diseases, in order to provide more experimental basis for clinical use and better serve the clinical.

## Materials and methods

### Antibodies and reagents

A mouse anti-srGAP3 antibody was purchased from Santa Cruz Biotechnology (Santa Cruz, CA, USA). A rabbit anti-PSD95 antibody, a rabbit anti-NMDAR2B (phospho Y1472) antibody and a mouse anti-NMDAR2B antibody were obtained from Abcam (Cambridge Science Park, Cambridge, UK). A mouse anti-active Rac1 antibody and the Rac1 Activity Assay Kit were obtained from New East (New East Biotechonology Co. Ltd, Wuhan, China). A rabbit anti-GAPDH antibody was purchased from Affinity Biosciences (Cincinnati, OH, USA). The rac1 activator CN04 was obtained from Cytoskeleton Inc. (Cat. CN04-A, Cytoskeleton, Colorado, USA). The FD Rapid GolgiStain Kit was obtained from FD Neurotechnologies, Inc (Guilford, MD, USA). The G-Actin/F-actin In Vivo Assay Kit was obtained from Cytoskeleton, Inc.

### Animals

Adult male Sprague–Dawley rats (200–250 g) were purchased from the Laboratory Animal Center of Wenzhou Medical University. The rats were placed in a stable environment (22–24 °C, 12/12 dark/light cycle) and were able to obtain food and water freely. Studies were approved by the Animal Research Committee of Wenzhou Medical University and followed the National Institutes of Health Guide for the Care and Use of Laboratory Animals.

### SNL

The process of inducing the SNL model was performed as described previously [25, 26]. Briefly, after the rats were anesthetized with 2% sodium pentobarbital (30 mg/kg, i.p.), the skin of the lower back was cut lengthwise, the right transverse process of the lumbar spine was removed, and the L5 spinal nerve was exposed to the visual field. Then the L5 spinal nerve was ligated with 4–0 silk thread. Rats in the sham group also underwent this operation, but the L5 nerve was not ligated.

### EA stimulation

The rats were fixed to the rat fixator that was independently developed by our laboratory (State Intellectual Property Office, patent application no. 201110021482.5), and EA treatment was performed in an awake state without anesthesia. The “Kunlun” (BL-60) and “Zusanli” (ST-36) acupoints were selected for this experiment. The rats were treated with EA (2 Hz, 1.5 mA) by an electric stimulation device (Hans-200e, Jisheng Medical Device) at an acupuncture depth of 2–3 mm. The treatment time started from the 11th day after modeling and was treated every other day for 30 min each time.

### Experimental design

First, the rats were randomized into a sham group (n = 18, sacrificed 28 days after SNL) and an SNL group (10 rats were sacrificed 3 days after SNL, 10 rats were sacrificed 7 days after SNL, 18 rats were sacrificed 10 days after SNL, 10 rats were sacrificed 14 days after SNL, 10 rats were sacrificed 21 days after SNL, 18 rats were sacrificed 28 days after SNL). Pain thresholds were also measured before SNL and at 3, 7, 10, 14, 21, and 28 days after SNL.

Next, the animals were treated with srGAP3 siRNA to evaluate the role of srGAP3 in EA-induced analgesia. Three srGAP3 siRNA sequences and a negative control siRNA were designed by Genepharma (Shanghai, China) (5′-3′): siRNA 1, sense, GCAUCAGUGCAGAAAGCAATT, antisense UUGCUUUCUGCACUGAUGCTT; siRNA 2, sense GCAGUAACCUCAUCACCAATT, antisense UUGGUGAUGAGGUUACUGCTT; siRNA 3, sense GCCUCAUACCAGCGAUGAATT, antisense UUCAUCGCUGGUAUGAGGCTT; and the negative control siRNA (siNC), sense UUCUCCGAACGUGUCACGUTT, antisense ACGUGACACGUUCGGAGAATT. The rats were randomized into an SNL group (n = 23), an SNL + siNC group (n = 23), an SNL + siRNA1 group (n = 23), an SNL + siRNA2 group (n = 10), and an SNL + siRNA3 group (n = 10). The siRNAs were dissolved in diethyl pyrocarbonate-treated water (20 pmol/μl). Rats in the SNL + siNC, SNL + siRNA1, SNL + siRNA2 and SNL + siRNA3 groups were administered a mixture of TurboFect in vivo transfection reagent (Thermo Scientific Inc.) (10 μl) and siRNAs (10 μl) via intrathecal injection every 2 days starting from day 11 after SNL. We found that intrathecal injection of srGAP3 siRNA1 could more inhibit the expression of srGAP3 in rats with SNL. Therefore, we used siRNA1 for the subsequent studies. Rats in the SNL + EA + siRNA (n = 18) group were administered siRNA1 30 min before EA treatment. Rats in the SNL and SNL + EA (n = 18) groups were intrathecally injected with equal volumes of vehicle at the same time.

Next, the role of Rac1 in EA-induced analgesia was examined. The rats were randomized into an SNL group (n = 18), an SNL + CN04 group (n = 18), an SNL + EA group (n = 18), and an SNL + EA + CN04 group (n = 18). Rats in the SNL + CN04 and SNL + EA + CN04 groups were intrathecally injected with CN04 (25 μg/ml, 10 μl) [4, 5] 30 min before EA treatment. Rats in the SNL and SNL + EA groups received equal volumes of saline during the same time period.

### Mechanical withdrawal threshold (MWT)

The MWT was examined by an electronic von Frey anesthesiometer (IITC Life Sciences, CA, USA) by an individual who was blinded to the experimental conditions, and the analysis time was relatively constant (13:00–16:00). The rats were placed in the instrument for 30 min to acclimatize, then von Frey stimulation was performed on the plantar surface of the foot until the foot was lifted, record the pain threshold. In total, we performed five such tests, with five-minute intervals. The maximum and minimum values were excluded, and the remaining data were averaged to obtain the final MWT [25, 26].

### Thermal withdrawal latency (TWL)

TWL was examined by an A37370 plantar tester (Ugo Basilee, Milan, Italy). After the rats were placed in the testing equipment for 30 min, the radiant heat of 58 °C was projected on the center of sole on the affected side through the glass plate. When the rats retracted or lifted their feet due to pain, the heat source was automatically turned off, the maximum tolerance time was displayed on the instrument, and this value was recorded. In total, we performed five such tests, with five-minute intervals. The maximum and minimum values were excluded, and the remaining data were averaged to obtain the final MWT [25, 26].

### Western blotting

L4-6 spinal cord segments were lysed in prepared protein lysis buffer (RIPA: PMSF = 100:1). The lysate was centrifuged, and the supernatant was collected. The protein concentration was examined by a BCA protein detection kit (Beyotime Corp, China). We added equal amounts of protein to a 10% Tris–HCl SDS-PAGE gel (Bio–Rad Laboratories, CA, USA) for electrophoresis at a voltage of 80 V. The current was then set to 300 mA to transfer the proteins to the PVDF membranes (Millipore Corp, MA, USA). After membrane transfer, we blocked the membranes in 5% skim milk for 2 h. The membranes were then placed into primary antibodies and incubated at 4 °C for 17–20 h. After being washed, the membranes were placed in secondary antibodies and incubated for 1–1.5 h. We used enhanced chemiluminescence (ECL) kits (Beyotime Corp, China) to visualize the membranes and used AlphaEaseFC (version 4.0) to perform the data analysis.

### Quantitative real-time PCR (qRT-PCR)

We used TRIzol reagent (Invitrogen, Carlsbad, CA, USA) to extract total RNA from the L4-6 spinal cord segments of rats. SYBR-Green Supermix (Tokyo, Japan) and a LightCycler 480 system (Roche, USA) were used for real-time PCR analysis. The primer sequences (5′-3′) were as follows: srGAP3, sense GAACAATGTCATCGTCCGCCTCTC, and antisense TGGTCACCTTCAGCAGCTCCTC; and beta-actin, sense TGTCACCAACTGGGACGATA, and antisense GGGGTGTTGAAGGTCTCAAA. The data were analyzed by the 2^−ΔΔCT^ method.

### Golgi staining and dendritic spine analysis

Tissues were stored in a mixed liquid of solution A and solution B (1:1) in dark for 14 days. Then tissues were placed in solution C and kept away from light for 2 days. Tissues were cut to 150 μm series slices. Then, we stained the sections according to the instructions.

The neurons used for the measurement and statistics of dendritic spines should follow the following principles: first, they should be located in the I-IV lamina of the dorsal horn of the spinal cord; Second, there must be obvious cell bodies; Third, neurons must be completely immersed and have a certain length of dendrites, and the primary dendrites may not require branches.

We classified dendritic spines according to the definitions described by Kim [12]. Briefly, when the neck structure was clear, the protrusion of the dendritic branches was considered to be the spine. If we did not observe a clear neck structure but did observe visible indentation on both sides of the junction between dendritic branches and processes, it was also defined as a dendritic spine. The neck of the dendritic spine originates from primary dendritic branches and is connected to the circular chamber constituting the head. The head is the main part that is in contact with presynaptic buttons [18]. In this study, we divided dendritic spines into two categories according to their shape: thin spines and mushroom spines. The head diameter of the thin spine was less than or equal to the length of the neck. The head diameter of the mushroom spine was larger than the length of the neck. There were three main reasons why we chose these two classifications. First, these two classification methods allowed us to classify dendritic spines using simple but strict rules. Second, these two classification methods excluded the identification of subtle structural changes in the shape, allowing us to collect a large number of samples and make the experiment more accurate. Third, many studies have used these two classification methods [21, 31]. We used ImageJ software to measure the head diameter, neck length and total length of dendritic spines. The number of dendritic spines/10 μm dendritic length was expressed here as the dendritic spine density.

### Rac1 activation assay

L4-6 spinal cord tissue was homogenized in a mixture of RIPA lysis buffer and protease inhibitor, and the supernatant was collected after centrifugation. Rac1 activity was examined using a Rac1 activity assay kit. Assay/lysis buffer and active Ras monoclonal antibodies were added to cell lysates, and a protein A/G gel column was thoroughly mixed by vortex oscillators. Then 20 ul of the suspended bead slurry was quickly removed and added to the centrifuge tubes. The tube was incubated at 4 °C for 1 h, gently shaken, and centrifuged, and the supernatant was discarded. The beads were washed three times with assay/lysis buffer and centrifuged, and the supernatant was discarded. The sample was resuspended with SDS-PAGE sample buffer. Then, Western blotting was performed. GTPγS- and GDP-treated proteins were used as positive and negative controls, respectively.

### F-actin/G-actin (F/G) ratio

The expression levels of F-actin and G-actin were examined by a G-Actin/F-actin In Vivo Assay Kit. First, 1000 µl of warm LAS2 per 100 mg (0.1 g) of tissue sample was added. The sample was homogenized with a homogenizer. The lysate was incubated at 37 °C for 10 min. The supernatants were pipetted into clearly labeled ultracentrifuge tubes. The samples were centrifuged at 100,000 × g and 37 °C for 1 h. Transfer the supernatant into a new test tube. Then, add F-actin depolymerization buffer to depolymerize actin. Next, 25 µl of 5 × SDS sample buffer was added to pellets and supernatant samples and mixed well. Then, Western blotting was performed as described above. The results were expressed as the F-actin/G-actin (F/G) ratio.

### Immunohistochemical staining

The spinal cord (L4-6) was fixed in 4% paraformaldehyde. The tissue was embedded with paraffin wax. The paraffin sections were dewaxed. The slices were incubated in 3% hydrogen peroxide for 25 min. The tissue was sealed with 3% BSA for 30 min and then diluted primary antibodies were added and incubated overnight. Then, the secondary antibodies (HRP-labeled) were added to the tissues and incubated for 50 min. Add DAB color developing solution and control the color developing time under the microscope. The positive color was brownish yellow, and the section was rinsed with running water to terminate the color development. Hematoxylin was added and incubated for 3 min, and then the sample was dehydrated and sealed. The nuclei were stained blue with hematoxylin, and positive staining with DAB was brownish yellow.

### Statistical analysis

SPSS 23.0 statistical software was used to analyze the data. The data were expressed as the mean ± SD. The pain threshold was analyzed by repeated measures two-way analysis of variance (ANOVA) with Bonferroni’s post-hoc tests. Other data were analyzed by one-way ANOVA with Dunnett’s post-hoc tests. A value of *p* < 0.05 was considered significant.

## Results

### The pain threshold decreased after SNL

**Fig. 1:**
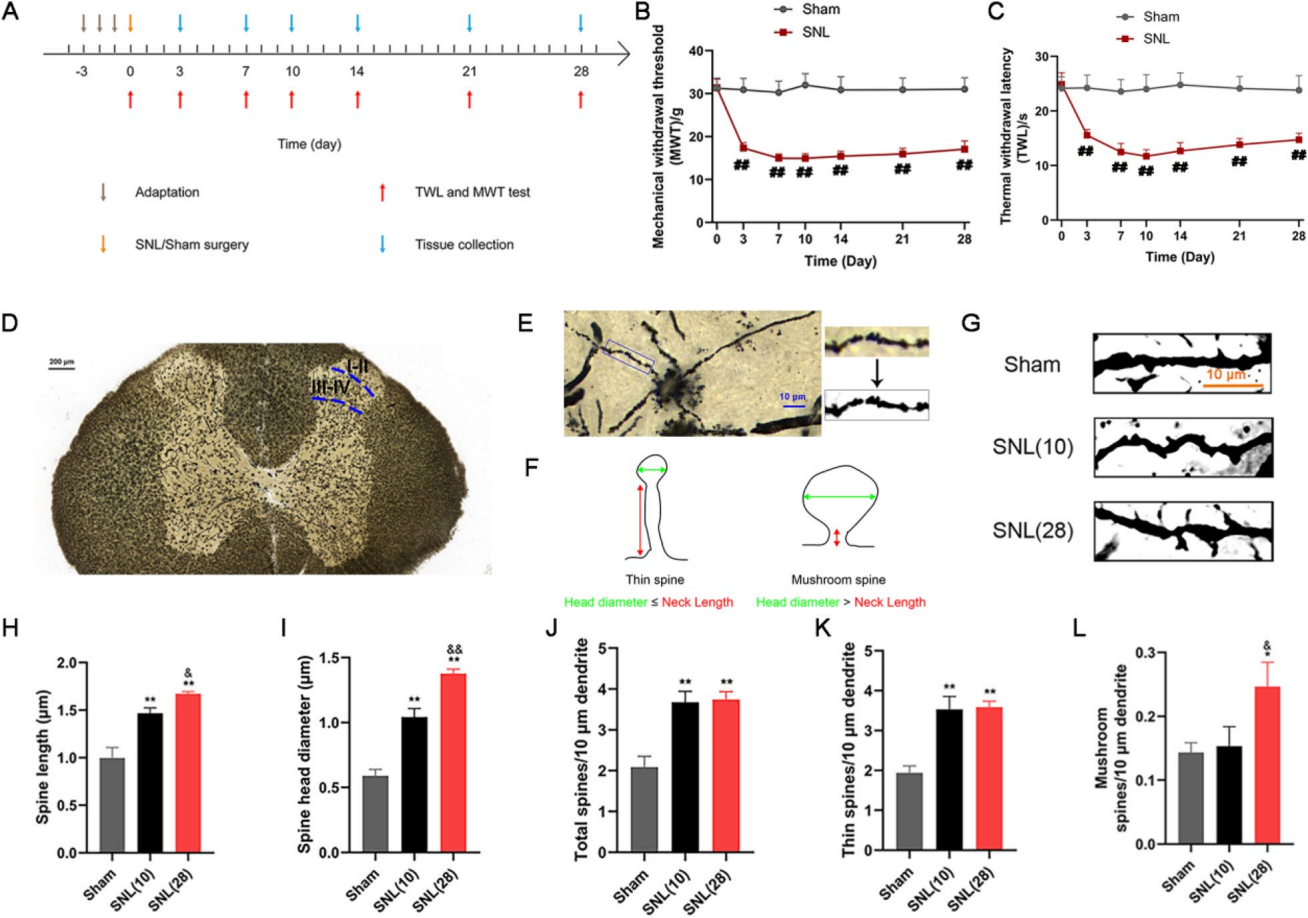
The pain threshold of rats decreased and the dendritic spines of the spinal dorsal horn were abnormally reconstructed after SNL. **A** Schematic diagram of the experimental process. **B** Mechanical withdrawal threshold (MWT). n = 15. ^##^*p* < 0.01 vs. the sham group. **C** Thermal withdrawal latency (TWL). n = 15. ^##^*p* < 0.01 vs. the sham group. **D**, **E** Example of dendritic spines of spinal dorsal horn neurons (the selected site of neurons was I-IV lamina). **F** Schematic diagram of the classification of thin and mushroom-shaped dendritic spines. **G** Dendritic spines. Scale bars, 10 μm. **H, I** Five neurons were randomly selected from the dorsal horn of the spinal cord of each rat, and 20 to 30 dendritic spines were randomly selected from each neuron, and the lengths and head diameters of dendritic spines were measured. n = 4. **J** Number of spines per 10 μm. n = 4. **K** Number of thin spines per 10 μm. n = 4. **L** Number of mushroom spines per 10 μm. n = 4. Columns represent the mean ± SD. ^*^p < 0.05, ^**^p < 0.01 vs the sham group; ^&^*p* < 0.05, ^&&^*p* < 0.01 vs. the SNL (10) group

The MWT and TWL were measured before SNL and on days 3, 7, 10, 14, 21 and 28 after SNL (Fig. 1B and C). Compared with those in the sham group, the MWT and TWL were significantly reduced in rats with peripheral nerve injury (*p* < 0.01).

### SNL induced dendritic spine remodeling in neurons of the spinal dorsal horn

On days 10 and 28 after SNL, the spinal dorsal horn tissues of rats were collected for Golgi staining (Fig. 1H and I). We found a significant increase in spine length and head diameter on day 10 compared to those in the sham group (*p* < 0.01). On day 28, compared with those in the sham group, spine length and head diameter increased significantly (*p* < 0.01). Compared with those on the 10th day, dendritic spines on the 28th day had longer lengths and larger head diameters (*p* < 0.05).

As shown in Fig. 1J–L, total spine density and thin-shaped spine density on days 10 and 28 increased after SNL compared with those in the sham group (*p* < 0.01). The density of total dendritic spines and thin-shaped dendritic spines on day 10 was similar to that on day 28 (*p* > 0.05). Compared with that in the sham group, the density of mushroom dendritic spines did not increase significantly on the 10th day (*p* > 0.05) but increased significantly on the 28th day (*p* < 0.05). The density of mushroom dendritic spines on the 28th day was higher than that on the 10th day (*p* < 0.05).

### The expression of srGAP3 increased gradually in the initial stage and decreased gradually in the later stage after SNL

**Fig. 2:**
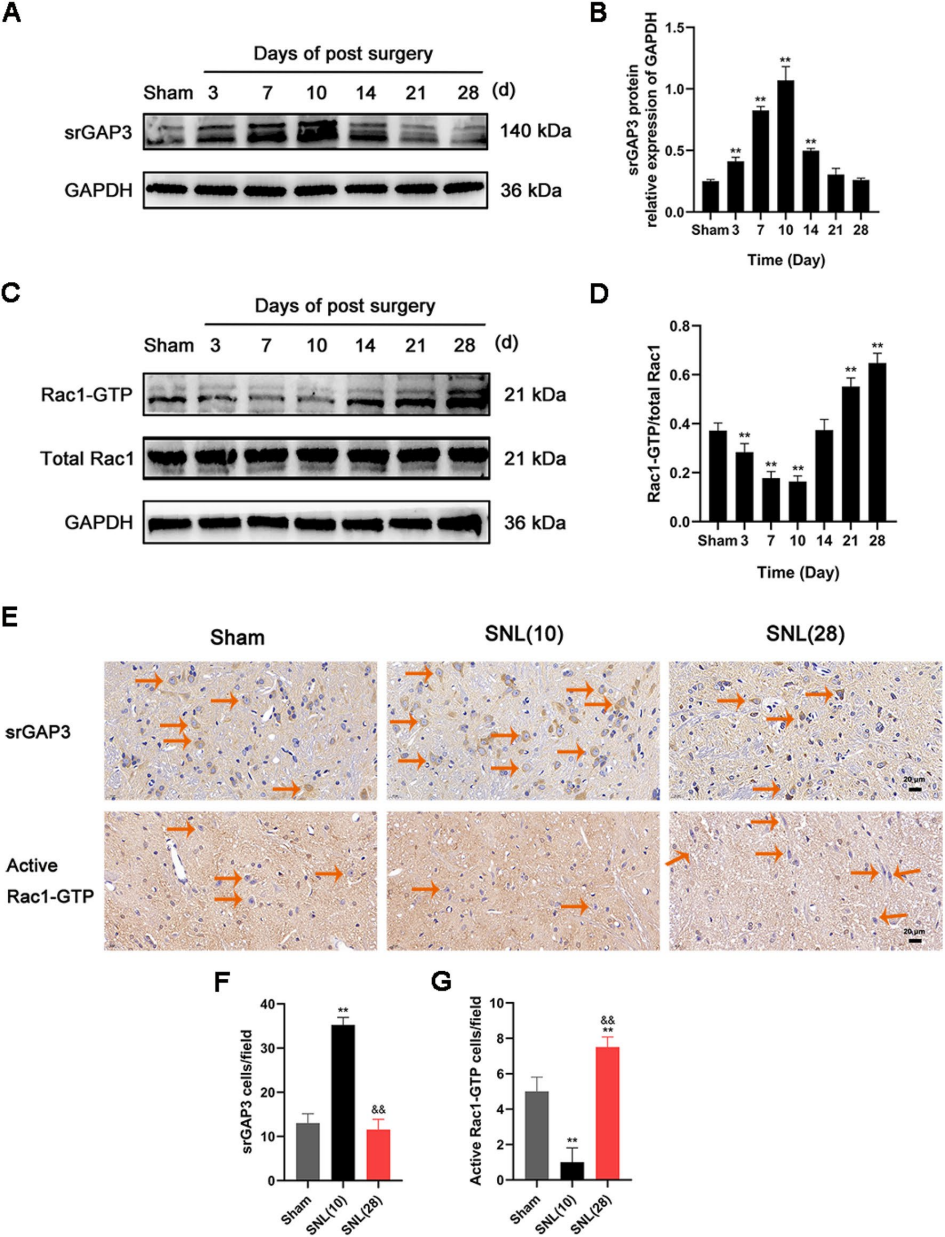
The expression of srGAP3 increased gradually in the initial stage of peripheral nerve injury and decreased gradually in the later stage, while the activity of Rac1 decreased gradually in the initial stage and increased gradually in the later stage after SNL. **A**, **B** Representative Western blots and quantitative analysis of srGAP3/GAPDH expression. n = 5. **C**, **D** Representative Western blots and quantitative analysis of Rac1-GTP/total Rac1 expression. n = 5. **E** Representative photos of immunohistochemical staining of srGAP3 and active Rac1-GTP cells (The orange arrows show some representative srGAP3- and active Rac1-GTP-positive cells). Scale bars, 20 μm. **F**, **G** Number of srGAP3- and active Rac1-GTP-positive cells per field in the spinal dorsal horn. n = 4. Columns represent the mean ± SD. ^**^p < 0.01 vs. the sham group; ^&&^*p* < 0.01 vs the SNL (10) group

We collected spinal dorsal horn L4-6 segments on the 3rd, 7th, 10th, 14th, 21st and 28th days after modeling. Western blotting (Fig. 2A and B) showed that the expression level of srGAP3 in spinal dorsal horn tissues increased gradually after SNL, peaked on day 10, and then gradually decreased.

Immunohistochemical analysis (Fig. 2E and F) showed that the number of srGAP3-positive cells in the spinal dorsal horn of rats on day 10 after modeling was higher than that in the sham group (*p* < 0.01). The number of srGAP3-positive cells on day 28 after modeling was less than that on day 10 (*p* < 0.01). There was no significant difference in the number of srGAP3-positive cells in the spinal dorsal horn in the sham group and on the 28th day after modeling (*p* > 0.05).

### The activity of Rac1 decreased gradually in the initial stage and increased gradually in the later stage after SNL

We collected spinal dorsal horn L4-6 segments from experimental rats on the 3rd, 7th, 10th, 14th, 21st and 28th days after SNL. Western blotting (Fig. 2C and D) showed that the activity of Rac1 decreased gradually after modeling, reached the lowest level on day 10, and then gradually increased.

Rac1-GTP represents the activity of Rac1. Immunohistochemical analysis (Fig. 2E and G) showed that the number of active Rac1-GTP-positive cells in the spinal dorsal horns of rats on day 10 after modeling was less than that in the sham group (*p* < 0.01). The number of active Rac1-GTP-positive cells on day 28 after modeling was greater than that in the sham group and on day 10 (*p* < 0.01).

### The expression of p-NR2B and PSD95 increased gradually after modeling and remained high in the later stage

**Fig. 3:**
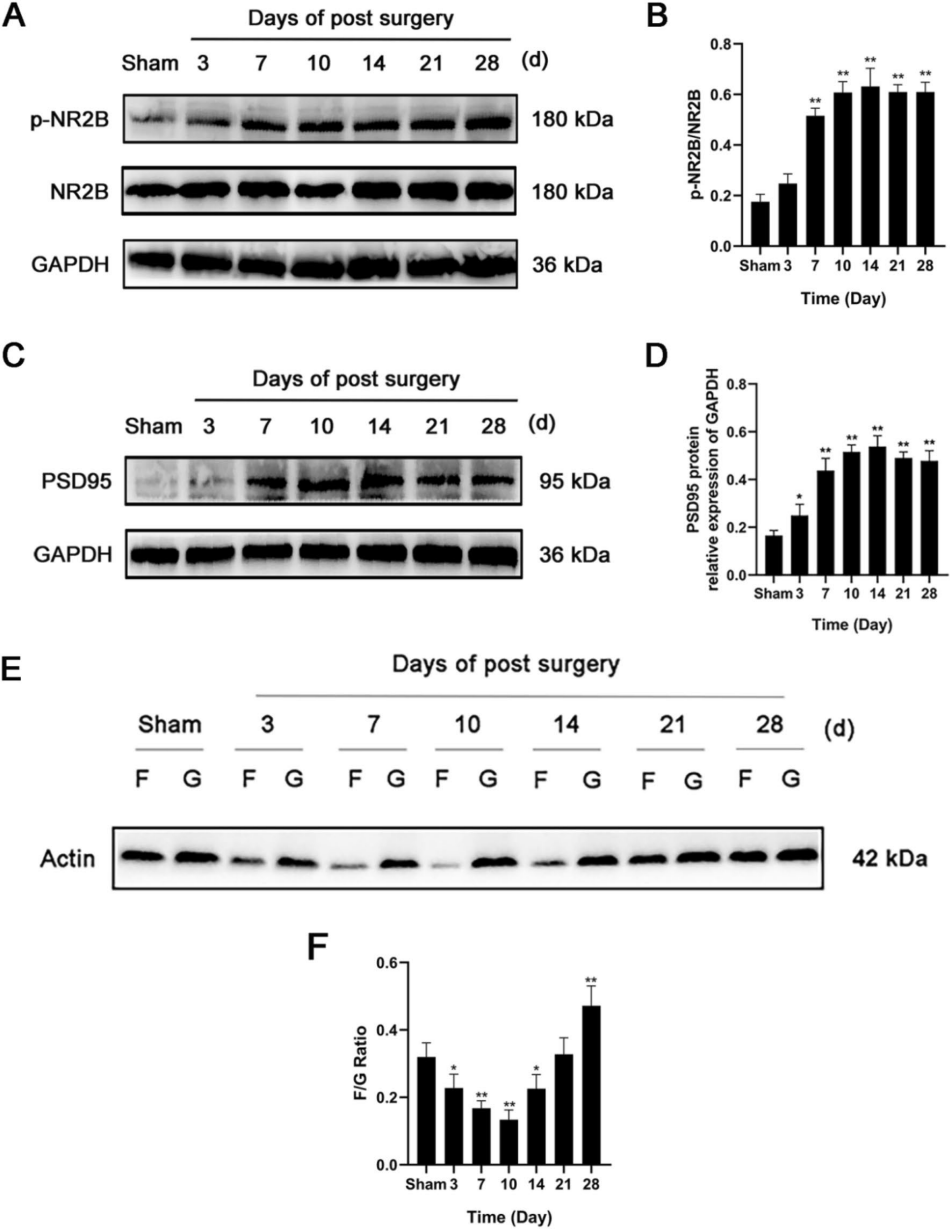
The expression of p-NR2B and PSD95 were increased after SNL, and the ratio of F/G decreased gradually in the initial stage and increased gradually in the later stage after SNL. **A**, **B** Representative Western blots and quantitative analysis of p-NR2B/NR2B expression. n = 5. **C**, **D** Representative Western blots and quantitative analysis of PSD95/GAPDH expression. n = 5. **E**, **F** Representative Western blots and quantitative analysis of the F/G ratio. n = 5. Columns represent the mean ± SD. ^*^p < 0.05 and ^**^p < 0.01 vs the sham group

L4-6 spinal cord segments were collected and examined by Western blotting on days 3, 7, 10, 14, 21 and 28 after modeling (Fig. 3A–D). The results showed that after modeling, the protein expression of p-NR2B and PSD95 increased gradually and remained high from the 7th day.

### The F/G ratio decreased gradually in the initial stage and increased gradually in the later stage after SNL

The levels of F-actin and G-actin in the L4-6 spinal cord segments of rats were measured on days 3, 7, 10, 14, 21 and 28 after modeling (Fig. 3E and F). The results showed that the F/G ratio gradually decreased during the initial stage after modeling, reached the lowest level on day 10, and then gradually increased.

### EA lowered mechanical and thermal hyperalgesia, inhibited abnormal dendritic spine remodeling, increased the expression of srGAP3 and decreased the activity of Rac1, the expression of p-NR2B and PSD95 and the ratio of F/G in rats with SNL, whereas srGAP3 siRNA abolished these effects

**Fig. 4:**
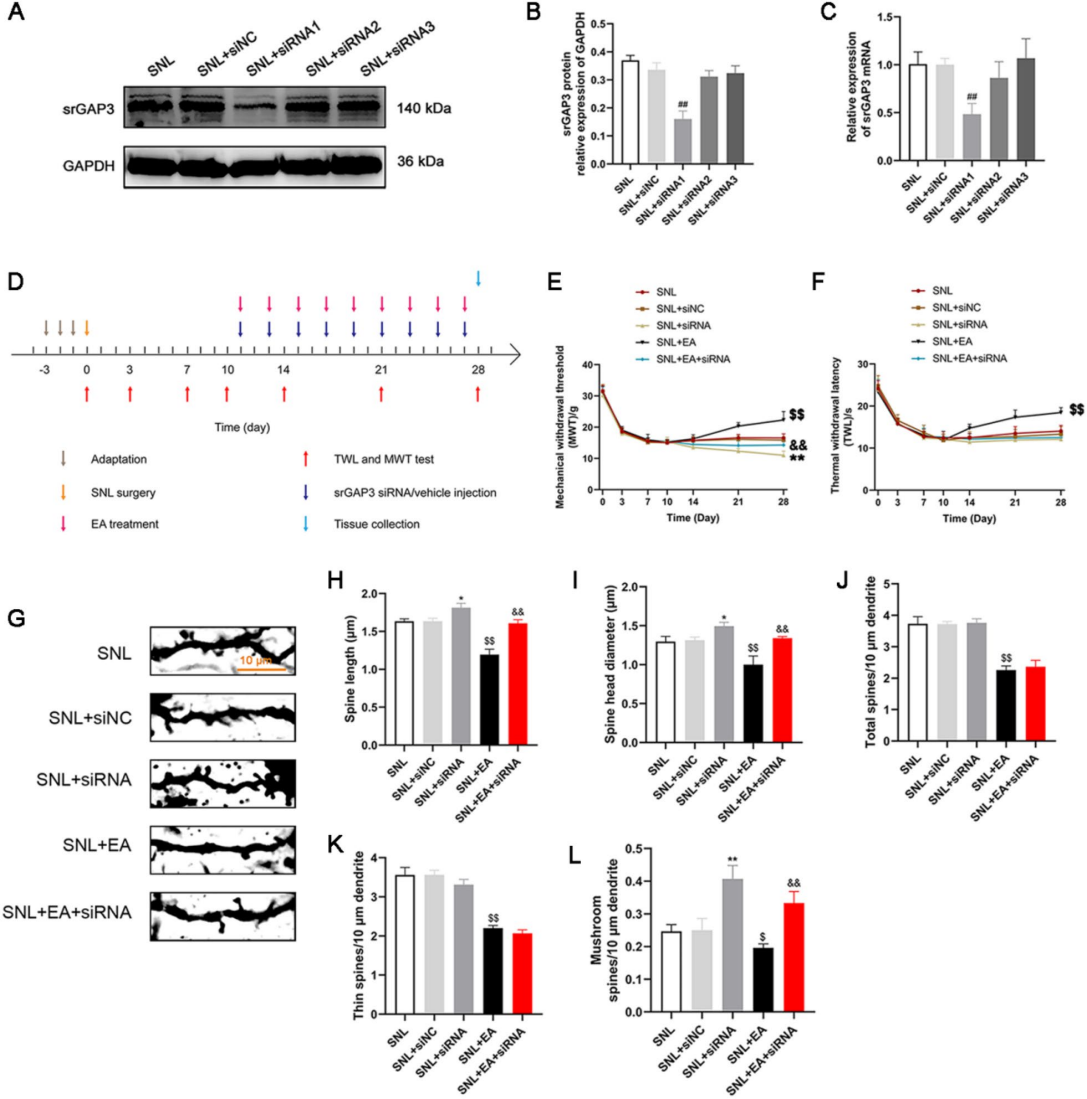
EA treatment increased the pain threshold and inhibited abnormal dendritic spine remodeling in spinal dorsal horn neurons in rats with SNL, whereas the use of srGAP3 siRNA reversed the effects of EA. **A**, **B** Representative Western blots and quantitative analysis of srGAP3/GAPDH expression. n = 5. Columns represent the mean ± SD. ^##^*p* < 0.01 vs. the SNL + siNC group. **C** The effects of siRNAs on the expression of srGAP3 mRNA examined by qRT–PCR. n = 5. Columns represent the mean ± SD. ^##^*p* < 0.01 vs. the SNL + siNC group. **D** Schematic diagram of the experimental process. **E** Mechanical withdrawal threshold (MWT). n = 15. ^$$^*p* < 0.01 vs. the SNL group, ^&&^*p* < 0.01 vs. the SNL + EA group, ^**^p < 0.01 vs. the SNL + siNC group. **F** Thermal withdrawal latency (TWL). n = 15. ^$$^*p* < 0.01 vs. the SNL group. **G** Dendritic spines. Scale bars, 10 μm. **H**, **I** Five neurons were randomly selected from the dorsal horn of the spinal cord of each rat, and 20 to 30 dendritic spines were randomly selected from each neuron, and the lengths and head diameters of dendritic spines were measured. n = 4. **J** Number of spines per 10 μm. n = 4. **K** Number of thin spines per 10 μm. n = 4. **L** Number of mushroom spines per 10 μm. n = 4. ^$^*p* < 0.05, ^$$^*p* < 0.01 vs. the SNL group, ^&&^*p* < 0.01 vs. the SNL + EA group, ^*^p < 0.05, ^**^p < 0.01 vs the SNL + siNC group

As shown in Fig. 4A–C, the protein and mRNA expression levels of srGAP3 were significantly decreased after siRNA1 was administered rats with SNL, while the other siRNAs designed for srGAP3 (siRNA2 and siRNA3) and siNC had no significant effect on the expression levels of srGAP3. Therefore, siRNA1 was selected for subsequent experiments.

The results of the behavioral test (Fig. 4E and F) showed that on the 28th day after modeling, MWT and TWL in the SNL + EA group was higher than that in rats in the SNL group (*p* < 0.01). MWT in the SNL + siRNA group was lower than that in the SNL + siNC group (*p* < 0.01), and TWL in the SNL + siRNA group was similar to that in the SNL + siNC group (*p* > 0.05). MWT in the SNL + EA + siRNA group was lower than that in the SNL + EA group (*p* < 0.01), and TWL in the SNL + EA + siRNA group was similar to that in the SNL + EA group (*p* > 0.05). There was no significant difference in TWL or MWT between the SNL and SNL + siNC groups (*p* > 0.05).

Golgi staining (Fig. 4G–L) showed that compared with those in the SNL group, the length, head diameter, total dendritic spine density, thin dendritic spine density and mushroom dendritic spine density of the spinal dorsal horn in the SNL + EA group decreased (*p* < 0.05). Compared with those in the SNL + siNC group, the length and head diameter of dendritic spines increased (*p* < 0.05), the density of mushroom dendritic spines increased (*p* < 0.01), and the total dendritic spine density and thin dendritic spine density did not change significantly in the SNL + siRNA group (*p* > 0.05). Compared with those in the SNL + EA group, the length of dendritic spines, head diameter, and density of mushroom dendritic spines in the SNL + EA + siRNA group increased (*p* < 0.01), while the total dendritic spine density and thin dendritic spine density did not change significantly (*p* > 0.05). There was no significant difference in dendritic spine length, head diameter or density between the SNL and SNL + siNC groups (*p* > 0.05).

**Fig. 5:**
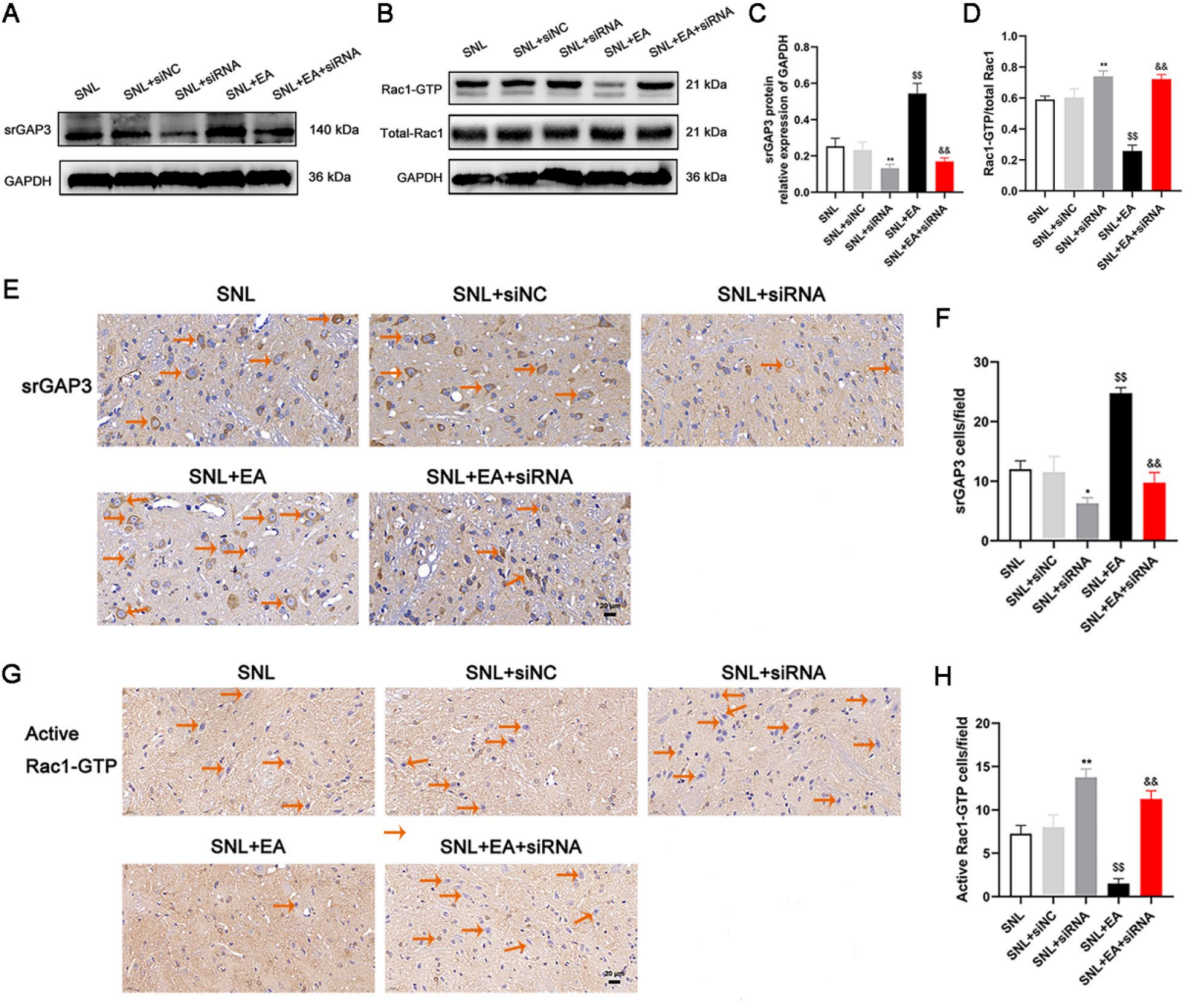
EA promoted the expression of srGAP3 and inhibited the expression of Rac1-GTP in the spinal dorsal horns of rats with SNL, while the use of srGAP3 siRNA reversed the effects of EA. **A–C** Representative Western blots and quantitative analysis of srGAP3/GAPDH expression. n = 5. **B**–**D** Representative Western blots and quantitative analysis of Rac1-GTP/total Rac1 expression. n = 5. **E**–**G** Representative photos showing immunohistochemical staining of srGAP3 and active Rac1-GTP cells (The orange arrows show some representative srGAP3- and active Rac1-GTP-positive cells). Scale bars, 20 μm. **F–H** Number of srGAP3- and active Rac1-GTP-positive cells per field in the spinal dorsal horn. n = 4. Columns represent the mean ± SD. ^$$^*p* < 0.01 vs. the SNL group, ^&&^*p* < 0.01 vs. the SNL + EA group, ^*^p < 0.05, ^**^p < 0.01 vs the SNL + siNC group

**Fig. 6:**
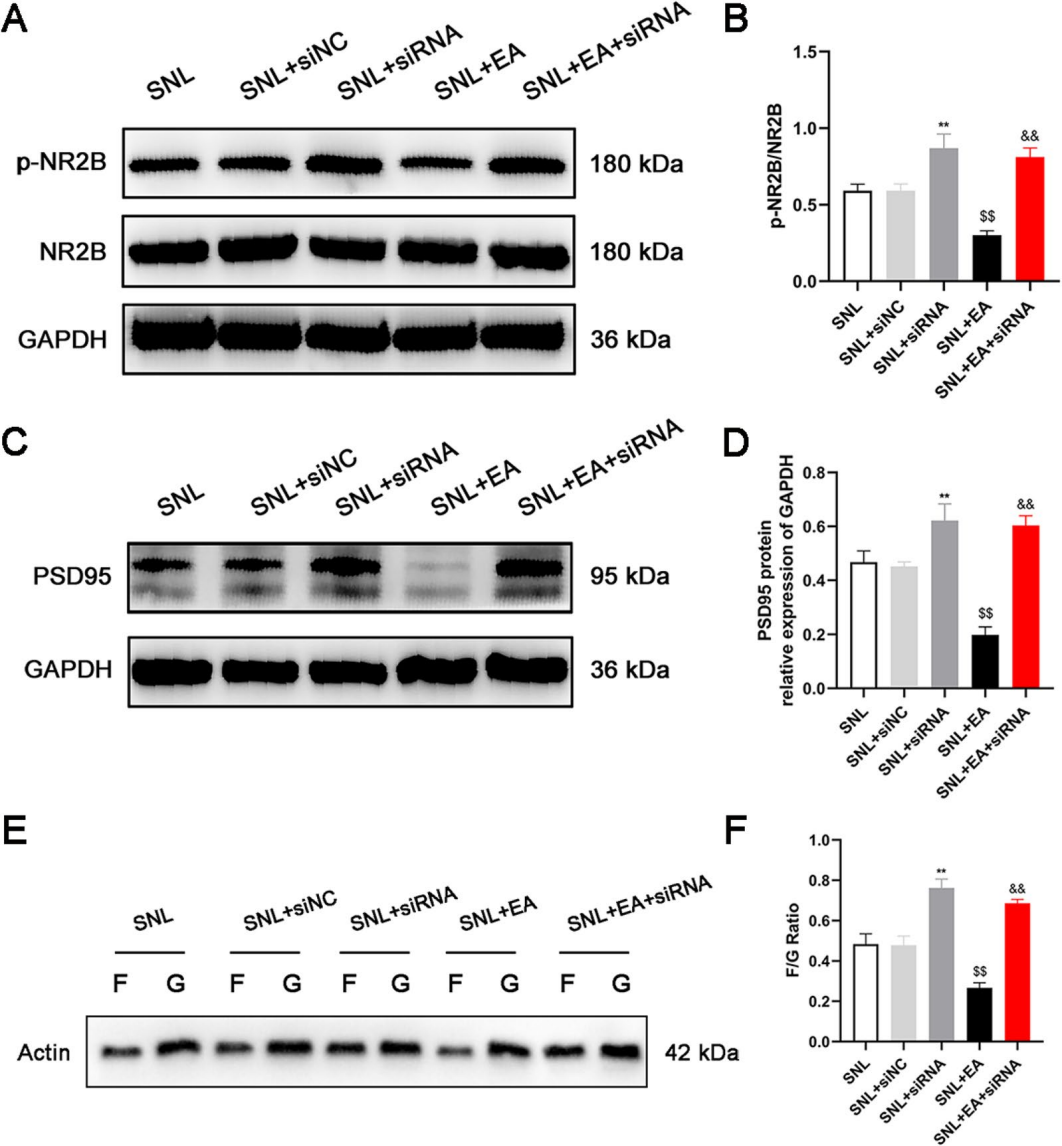
EA decreased the expression of p-NR2B and PSD95 and the ratio of F/G in the spinal dorsal horns of rats with SNL, while the use of srGAP3 siRNA reversed the effects of EA. **A**, **B** Representative Western blots and quantitative analysis of p-NR2B/NR2B expression. n = 5. **C**, **D** Representative Western blots and quantitative analysis of PSD95/GAPDH expression. n = 5. **E**, **F** Representative Western blots and quantitative analysis of the F/G ratio. n = 5. ^$$^*p* < 0.01 vs. the SNL group, ^&&^*p* < 0.01 vs. the SNL + EA group, ^**^p < 0.01 vs. the SNL + siNC group

Western blotting (Figs. 5A–D and 6A–D) showed that compared with that in the SNL group, the expression of srGAP3 in the spinal dorsal horn in the SNL + EA group increased (*p* < 0.01), and the expression of Rac1-GTP/total Rac1, p-NR2B/NR2B and PSD95 decreased (*p* < 0.01). Compared with that in the SNL + siNC group, the expression of srGAP3 decreased (*p* < 0.01), and the expression of Rac1-GTP/total Rac1, p-NR2B/NR2B and PSD95 increased in the SNL + siRNA group (*p* < 0.01). Compared with that in the SNL + EA group, the expression of srGAP3 decreased, and the expression of Rac1-GTP/total Rac1, p-NR2B/NR2B and PSD95 increased in the SNL + EA + siRNA group (*p* < 0.01). There was no significant difference in srGAP3, Rac1-GTP/total Rac1, p-NR2B/NR2B or PSD95 expression between the SNL and SNL + siNC groups (*p* > 0.05). Changes in the expression of srGAP3 and active Rac1-GTP were examined by immunohistochemistry (Fig. 5E–H) in each group and were consistent with those of srGAP3 and active Rac1-GTP, as shown by Western blotting.

The F/G test results (Fig. 6E and F) showed that EA treatment reduced the F/G ratio in rats with SNL (*p* < 0.01). Compared with that in the SNL + siNC group, the F/G ratio in the SNL + siRNA group was higher (*p* < 0.01). A higher F/G ratio was observed in the SNL + EA + siRNA group than in the SNL + EA group (*p* < 0.01). There was no significant difference in the F/G ratio between the SNL and SNL + siNC groups (*p* > 0.05).

### EA increased the pain threshold, inhibited abnormal dendritic spine remodeling, and decreased the activity of Rac1, the expression of p-NR2B and PSD95 and the ratio of F/G in rats with SNL, while CN04 prevented these effects

**Fig. 7:**
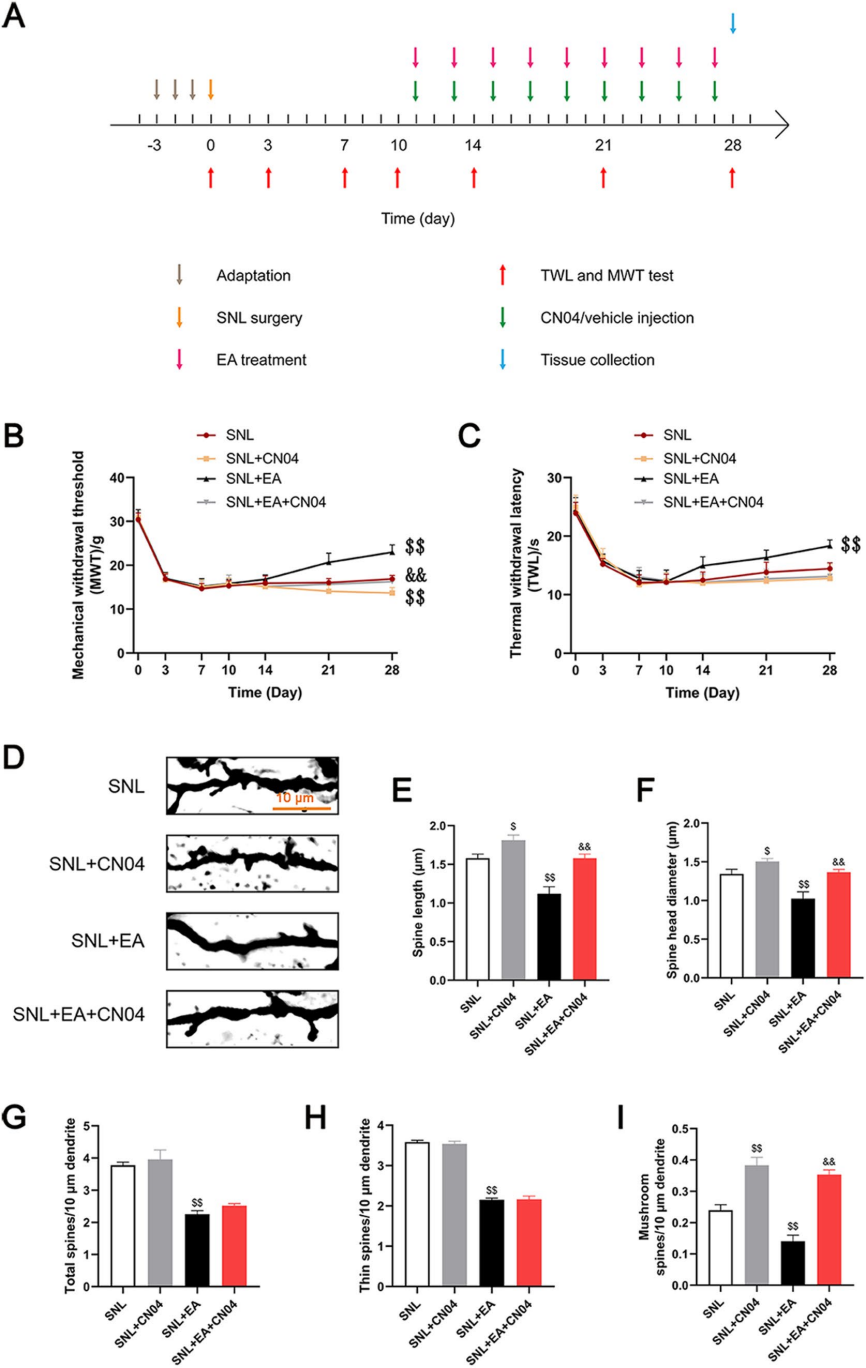
EA treatment increased the pain threshold and inhibited abnormal dendritic spine remodeling in rats with SNL, while CN04 reversed the effect of EA on the MWT and synaptic remodeling. **A** Schematic diagram of the experimental process. **B** Mechanical withdrawal threshold (MWT). n = 15. **C** Thermal withdrawal latency (TWL). n = 15. **D** Dendritic spines. Scale bars, 10 μm. **E**, **F** Five neurons were randomly selected from the dorsal horn of the spinal cord of each rat, and 20 to 30 dendritic spines were randomly selected from each neuron, and the lengths and head diameters of dendritic spines were measured. n = 4. **G** Number of spines per 10 μm. n = 4. **H** Number of thin spines per 10 μm. n = 4. **I** Number of mushroom spines per 10 μm. n = 4. ^$^*p* < 0.05, ^$$^*p* < 0.01 vs the SNL group, ^&&^*p* < 0.01 vs. the SNL + EA group

The results of the behavioral test (Fig. 7B and C) showed that on the 28th day after SNL, the pain threshold in rats in the SNL + EA group was higher than that in rats in the SNL group (*p* < 0.01). Compared with that in the SNL group, the mechanical threshold in the SNL + CN04 group decreased (*p* < 0.01), and the thermal threshold did not change significantly (*p* > 0.05). Compared with that in the SNL + EA group, the mechanical threshold in the SNL + EA + CN04 group was lower (*p* < 0.01), and the thermal threshold in the SNL + EA + CN04 group was similar to that in the SNL + EA group (*p* > 0.05).

The Golgi staining results (Fig. 7D–I) showed that the use of CN04 increased the length, head diameter and mushroom dendritic spine density in the spinal dorsal horns of rats with SNL (*p* < 0.05), but the change in total dendritic spine density and thin dendritic spine density was not obvious (*p* > 0.05). Compared with those in the SNL group, the length, head diameter, total dendritic spine density, thin dendritic spine density and mushroom dendritic spine density of the spinal dorsal horn in the SNL + EA group decreased (*p* < 0.01). Compared with those in the SNL + EA group, the length, head diameter, and mushroom dendritic spine density of the spinal dorsal horn in the SNL + EA + CN04 group increased (*p* < 0.01), while the total and thin dendritic spine densities in the SNL + EA + CN04 group did not change significantly (*p* > 0.05).

**Fig. 8:**
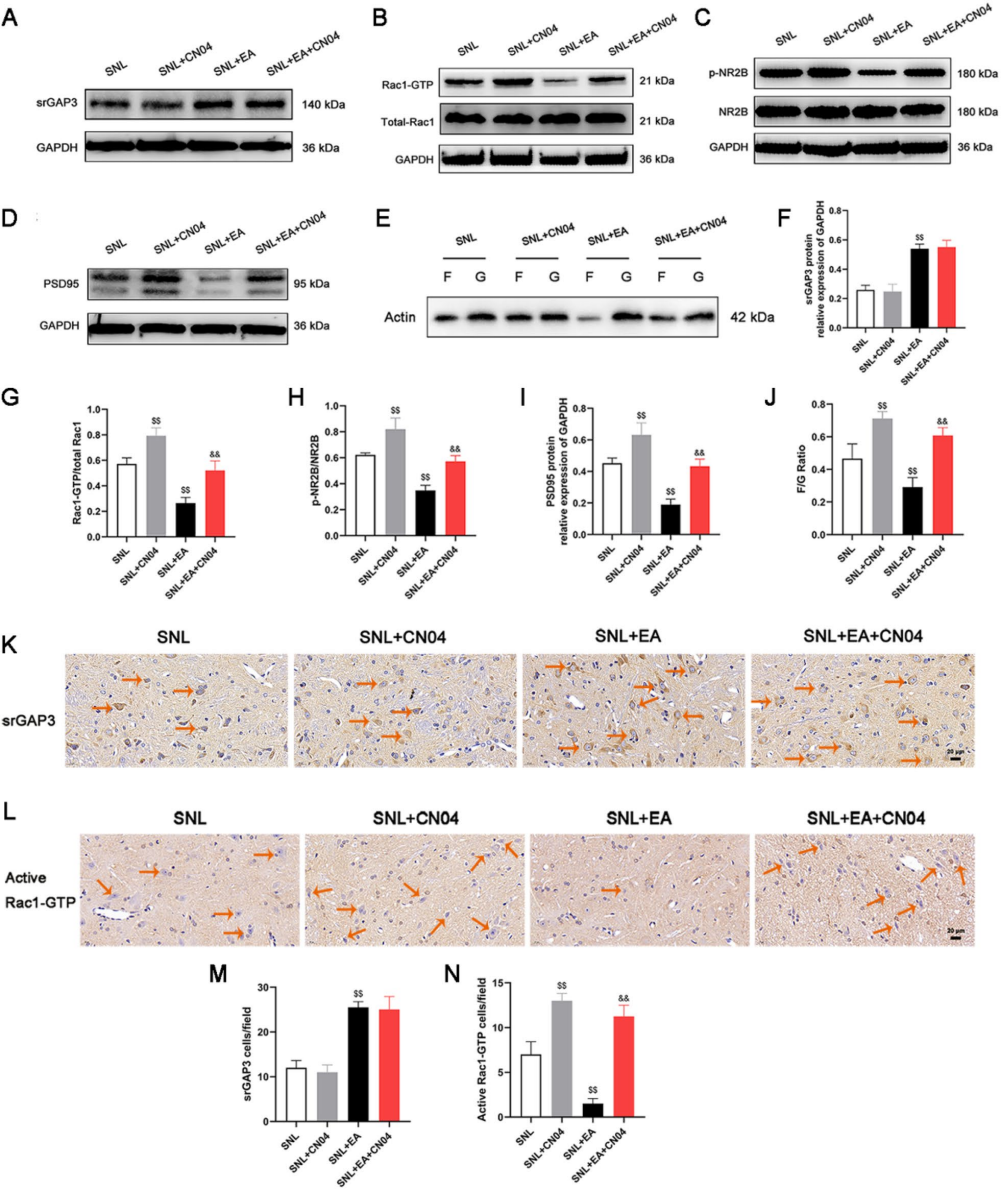
EA promoted the expression of srGAP3 and inhibited the expression of Rac1 GTP, p-NR2B, PSD95 and the ratio of F/G in the spinal dorsal horns of rats with SNL, while CN04 reversed the effects of EA on the expression of Rac1 GTP, p-NR2B, PSD95 and the ratio of F/G but had no significant effect on the expression of srGAP3. **A–F** Representative Western blots and quantitative analysis of srGAP3/GAPDH expression. n = 5. **B–G** Representative Western blots and quantitative analysis of Rac1-GTP/total Rac1 expression. n = 5. **C**–**H** Representative Western blots and quantitative analysis of p-NR2B/NR2B expression. n = 5. **D–I** Representative Western blots and quantitative analysis of PSD95/GAPDH expression. n = 5. **E–J** Representative Western blots and quantitative analysis of the F/G ratio. n = 5. **K**, **L** Representative photos showing immunohistochemical staining of srGAP3 and active Rac1-GTP cells (The orange arrows show some representative srGAP3- and active Rac1-GTP-positive cells). Scale bars, 20 μm. **M**, **N** Number of srGAP3- and active Rac1-GTP-positive cells per field in the spinal dorsal horn. n = 4. ^$$^*p* < 0.01 vs. the SNL group, ^&&^*p* < 0.01 vs the SNL + EA group

Western blotting (Figs. 8A–D and  8F–I) showed that the use of CN04 had no effect on the expression of srGAP3 in rats with SNL (*p* > 0.05) but promoted the expression of Rac1-GTP/total Rac1, p-NR2B/NR2B and PSD95 (*p* < 0.01). Compared with that in the SNL group, the expression of srGAP3 in the SNL + EA group increased (*p* < 0.01), and the expression of Rac1-GTP/total Rac1, p-NR2B/NR2B and PSD95 decreased (*p* < 0.01). Compared with that in the SNL + EA group, the expression of Rac1-GTP/total Rac1, p-NR2B/NR2B and PSD95 increased in the SNL + EA + CN04 group (*p* < 0.01). There was no significant difference in srGAP3 expression between the SNL + EA and SNL + EA + CN04 groups (*p* > 0.05). Changes in the expression of srGAP3 and active Rac1-GTP were examined by immunohistochemistry (Fig. 8K–N) and were consistent with those of srGAP3 and active Rac1-GTP, as examined by Western blotting.

The F/G test results (Fig. 8E and J) showed that the use of CN04 increased the F/G ratio in rats with SNL (*p* < 0.01). Compared with that in the SNL group, the F/G ratio in the SNL + EA group was lower (*p* < 0.01). A higher ratio of F/G was observed in the SNL + EA + CN04 group than in the SNL + EA group (*p* < 0.01).

## Discussion

In this study, we found that srGAP3 and Rac1 have different manifestations in different stages after SNL, and srGAP3 plays an important regulatory role in Rac1. In addition, EA can relieve neuropathic pain by regulating the plasticity of dendritic spines in the spinal dorsal horns of rats with peripheral nerve injury, and the srGAP3/Rac1 signaling pathway plays an important role in this process.

The core feature of neuropathic pain is the hyperexcitability of central nervous system and peripheral nervous system (including dorsal root ganglion and spinal dorsal horn) in nociceptive pathway. There is increasing evidence that there is a strong correlation between dendritic spine remodeling and pain caused by injury or disease [9, 31]. Dendritic spines are small processes on the dendrites of neurons, which form the postsynaptic components of most excitatory synapses. These structures play a key role in synaptic transmission and plasticity. Even small shape changes can strongly affect excitatory postsynaptic potentials and cause changes in action potentials [20]. The latest research with molecular imaging technology shows that dendritic spines are complex and dynamic structures that contain a dense cytoskeleton and transmembrane and scaffold molecules [1]. Some neurological and psychiatric diseases show abnormal dendritic spine structure and function. Pathologically, the number and size of dendritic spines changes due to injury and disease [1]. Pharmacological inhibitors that block dendritic spine remodeling can reduce dendritic spine hypoplasia and neuropathic pain caused by injury. These studies show that the analysis of dendritic spine plasticity is critical in the treatment of neuropathic pain. In our study, Golgi staining showed that the length and head diameter of dendritic spines in the dorsal horn of the spinal cord increased gradually after peripheral nerve injury. Spines with longer lengths and larger heads tended to have more glutamate receptors, increased PSD, and increased signaling efficiency. Moreover, we found that the density of dendritic spines in the dorsal horn of the spinal cord in rats with SNL was increased in the initial stage after peripheral nerve injury, but these spines were not yet mature, indicating that the newly formed immature dendritic spines could also improve the efficiency of interneuron signal transmission and promote hyperalgesia to a certain extent. The density of dendritic spines in the later stage of injury was similar to that in the initial stage, but the spines were more mature. The increase in mushroom dendritic spines indicated the enhancement of synaptic efficiency and stability. During the occurrence and development of neuropathic pain, the formation, extension and remodeling of dendritic spines parallel the natural development of dendritic spines.

EA plays an important role in regulating neuropathic pain. Studies have shown that EA can regulate synaptic plasticity by upregulating the expression of basic fibroblast growth factor to inhibit neuropathic pain caused by SNL [33]. EA stimulation of the acupoints “Zusanli” and “Kunlun” inhibited excitatory postsynaptic potential and reduced the efficiency of excitatory transmission between synapses, thus increasing the pain threshold of rats with peripheral nerve injury [32]. EA can also relieve neuropathic pain by regulating neuronal plasticity [25, 26]. However, the regulatory effect of EA on the dendritic spines of spinal cord neurons in rats with neuropathic pain remains to be further studied. The “Kunlun” (BL-60) and “Zusanli” (ST-36) points are commonly used for the treatment of lower limb pain [25, 26, 29]. Previous studies have also shown that 2 Hz of EA has a good analgesic effect [27]. As shown in this study, EA treatment increased the pain threshold of rats with SNL and alleviated neuropathic pain. EA treatment inhibited the SNL-induced increase in dendritic spine length, head diameter and dendritic spine density in the dorsal horn of the spinal cord. These results showed that EA plays an important role in regulating the plasticity of dendritic spines in the dorsal horn of the spinal cord in rats with peripheral nerve injury, but the specific mechanism needs to be further explored.

Studies have shown that srGAP3 is involved in the initial stage of neuropathic pain. The increase in srGAP3 can promote the formation of immature dendritic spines [24]. Moreover, some studies have shown that srGAP3 can negatively regulate the activity of Rac1 [4, 5]. Rac1 is a key regulator of actin and plays an important role in the formation, maintenance and dendritic spine remodeling [22]. Rac1 can also regulate the expression of NR2B and affect the occurrence and development of neuropathic pain [4, 5]. Knockout of NR2B inhibited NMDA receptor-dependent LTP and reduced dendritic ridge density [13]. PSD-95 mostly accumulates in excitatory synapses and interacts with synaptic receptors. The interaction between PSD-95 and NR2B contributes to NR2B phosphorylation and regulates NMDA receptor function [14]. Based on these studies, we hypothesized that the changes in the structure and function of dendritic spines in rats with SNL may be related to the srGAP3-Rac1 signaling pathway. In this study, we found that the expression level of srGAP3 gradually increased in the initial stage after SNL and gradually decreased in the later stage. The activity of Rac1 gradually decreased in the initial stage after SNL but gradually increased in the later stage, which was consistent with the efficiency of the conversion of soluble G-actin into filamentous F-actin, suggesting that srGAP3 and Rac1 may regulate dendritic spine maturity by regulating the actin network in neurons. Thus, the pain sensitivity of rats with peripheral nerve injury was affected. Analysis of PSD-95 and NR2B showed that the expression level of PSD-95 and the phosphorylation level of NR2B increased gradually after SNL and remained high in the later stage, which was consistent with the previously reported high levels of PSD-95 and p-NR2B in rats with neuropathic pain [15]. SrGAP3 siRNA and CN04 were administered to rats with SNL at the late stage of pain and further reduced the MWT, promoted the activity of Rac1, increased the expression of PSD-95 and the phosphorylation level of NR2B, and increased the ratio of F/G, but CN04 did not affect the expression of srGAP3. These results indicate that srGAP3 has regulatory effect on Rac1 and actin remodeling. The expression levels of PSD-95 and p-NR2B gradually increased in the early stage, which may be due to the increase in srGAP3 in the early stage, which promoted their expression. However, although srGAP3 gradually decreased in the late stage, the activity of Rac1 gradually increased, which maintained the high expression levels of PSD-95 and p-NR2B. This finding is consistent with the persistence of pain. In addition, the use of srGAP3 siRNA and CN04 promoted an increase in dendritic spine length and head diameter in rats with SNL, and promoted an increase in mushroom dendritic spine numbers, but had no significant effect on the total dendritic spine density or thin dendritic spine density, indicating that srGAP3 and Rac1 are mainly responsible for regulating the maturity of dendritic spines in the later stage of neuropathic pain. These findings indicate that the srGAP3/Rac1 pathway can affect neuronal excitability by regulating the activity of the PSD region and actin network, thus mediating the occurrence and development of pain.

We further analyzed the relationship between the therapeutic effect of EA and the srGAP3/Rac1 signaling pathway. EA treatment increased the mechanical threshold and thermal threshold of rats with SNL, but the use of srGAP3 siRNA and CN04 only reversed the EA-mediated increase in mechanical threshold and had no effect on EA-mediated inhibition of thermal sensitivity. These results showed that EA affected the mechanical threshold of rats with SNL through the srGAP3/Rac1 signaling pathway, but the inhibitory effect of EA on thermal sensitivity did not occur through the srGAP3/Rac1 signaling pathway. Furthermore, EA promoted srGAP3 expression, inhibited Rac1 activity, reduced PSD-95 and p-NR2B expression and decreased the F/G ratio in rats with SNL, while the use of srGAP3 siRNA and CN04 reversed these effects of EA. These results indicate that EA regulates actin remodeling through srGAP3/Rac1 signal pathway, thus exerting analgesic effect. Longer spine lengths and larger spine head diameters were observed in the SNL + EA + siRNA or SNL + EA + CN04 groups than in the SNL + EA group. However, the use of srGAP3 siRNA and CN04 only reversed the EA-mediated reduction in mushroom dendritic spine density and did not affect the reduction in total dendritic spine density or thin dendritic spine density. These results suggest that EA plays a role in the late stage of neuropathic pain by regulating the maturity of dendritic spines through the srGAP3/Rac1 pathway, but the mechanism by which EA inhibits the formation of new dendritic spines remains to be further studied.

**Fig. 9:**
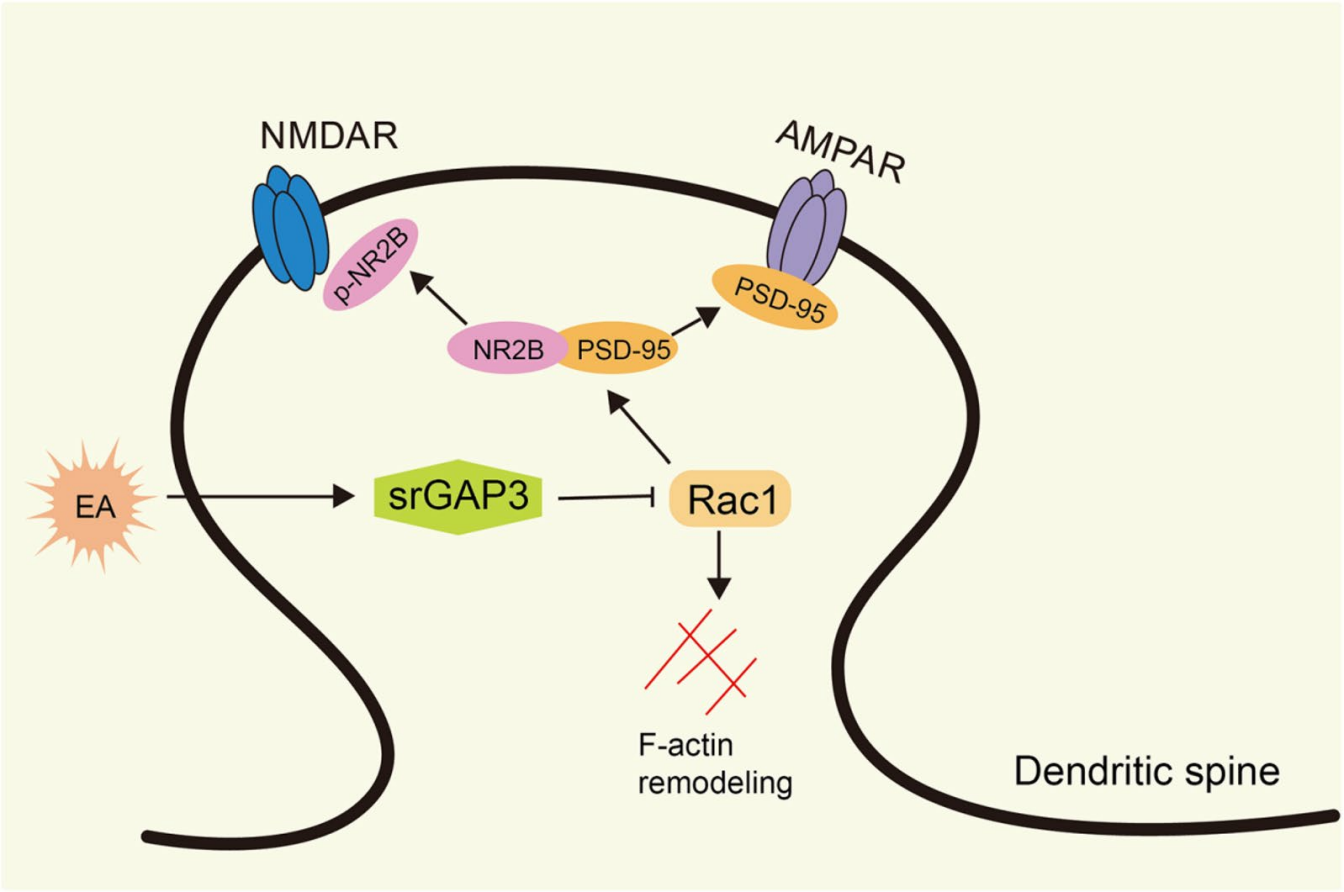
Possible mechanism for the analgesic effect of EA after SNL injury. In the late stage of peripheral nerve injury, EA inhibits the activity of Rac1 by promoting the expression of srGAP3 in spinal dorsal horn, thus regulating the activity of the PSD region and actin network of dendritic spine, and thus alleviating neuropathic pain

In conclusion, the findings of this study suggest that EA can inhibit abnormal dendritic spine remodeling in the spinal dorsal horns of rats with peripheral nerve injury through the srGAP3/Rac1 signaling pathway and increase the mechanical pain threshold (Fig. 9). Therefore, EA plays an important role in alleviating neuropathic pain, but other mechanisms related to EA-induced analgesia remain to be clarified.

## Data Availability

The datasets used and/or analysed during the current study are available from the corresponding author on reasonable request.
